# Traditions, beliefs and indigenous technologies in connection with the edible longhorn grasshopper *Ruspolia differens* (Serville 1838) in Tanzania

**DOI:** 10.1186/s13002-017-0191-6

**Published:** 2017-11-13

**Authors:** Mercy W. Mmari, John N. Kinyuru, Henry S. Laswai, Judith K. Okoth

**Affiliations:** 1 Department of Food Science and Technology, Jomo Kenyatta University of Agriculture and Technology, P. O. Box 62000-00200, Nairobi, Kenya; 2 Department of Food Technology, Nutrition and Consumer Sciences, Sokoine University of Agriculture, P.O. Box 3006, Chuo Kikuu, Morogoro, Tanzania

**Keywords:** Senene, Edible insects, Indigenous processing, Tanzania, Ethno-entomology, Food, Grasshopper

## Abstract

**Background:**

Edible insects are an important source of food to many African populations. The longhorn grasshopper, *Ruspolia differens* (Serville 1838), commonly known as *senene* in Tanzania is one of the most appreciated edible insects by societies around Lake Victoria crescent. *Senene* is primarily an essential treat for the tribes around the lake, e.g., the *Haya* of Tanzania, Luo of Kenya and Baganda of Uganda. Despite its importance as a food item and appreciation as a delicacy, there are few studies dealing with culture, beliefs and indigenous technology in connection with the *senene*. The main objective of this study was to survey indigenous technologies, processing methods and traditions in relation to *senene* consumption among the Haya tribe in Kagera region of Tanzania.

**Methods:**

Our ethnographic study was conducted through semi-structured interviews. A total of 51 locals, 26 females and 25 males aged 21 to 60 years were interviewed (with 3 female and 7 male key informants among them). Questions focused on cultures, beliefs and traditions towards *senene* consumption. Processing, preservation and shelf-life as well as nutritional knowledge were also investigated.

**Results:**

Harvesting for household consumption was mainly done through wild collection. Traditionally made traps were mostly used for commercial harvesting. Deep frying was the most preferred processing method while smoking was the most preferred preservation method, with shelf-life of up to 12 months. Interesting traditions and taboos associated with *senene* consumption were identified, with men monopolising the insects as food by declaring the insects taboo for women and children. Deep fried *senene* in locally packed containers were mostly sold by street vendors, but also available from a variety of stores and supermarkets.

**Conclusion:**

Beyond being just an important traditional delicacy, *senene* is becoming increasingly popular, providing opportunity for local businesses. Indigenous technologies for harvesting, processing and preserving *senene* exist, but must be improved to meet food processing standards, thereby promoting commercialization. This carries economic potential essential for improving incomes and livelihoods of women and smallholder farmers, improving household level food security.

## Background

Anthropo-entomophagy is spreading globally [1]. Although not fully embraced by the majority of Western cultures, consumption of edible insects by humans (anthropo-entomophagy) has existed since ancient times [2, 3]. In developed countries like Japan and Korea, the grasshopper, *Oxya yezoensis* [4], remains popular as an edible species and globally, Orthoptera like grasshoppers and katydids, e.g., *Ruspolia differens* [5], locusts like *Locusta migratoria* [6] and crickets [7] play a major role among the more than 2000 species currently considered edible [8].

The East African longhorn grasshopper (*R. differens*) is a cone-headed species commonly known as *senene* in Swahili which belongs to the family Tettigoniidae, suborder Ensifera and order Orthoptera. It is easily confused with related species including *Ruspolia lineosa*, *Ruspolia nitidula* and *Ruspolia dubia* [9] who are also referred to as *senene*. *R. differens* has different local names and is known as *senesene* in Kenya [10], *nsenene* in Uganda [9] and *nshokonono* in Zambia [11]. The nomenclature of *R. differens* has been a subject of interest among entomologists since its first description by Serville in 1838 as cited in [12]. More than ten scientific names have been suggested since then [9, 13–15].

*Senene* has been widely harvested and consumed as a traditional snack in Zambia and regions around Lake Victoria crescent including Tanzania, Uganda, Kenya and Democratic Republic of Congo. In these areas, indigenous people customarily consider *senene* and other edible insects to be highly nutritious and believe they have specific medicinal properties. The culture of *senene* consumption is indigenous to the *Haya* tribe found in the Lake zone of Tanzania, where the offering of *senene* is seen as a sign of respect to the person it is served to. This culture has now spread throughout Tanzania where *senene* is commercially available and widely consumed by different tribes. *Senene* is reported to be nutritious consisting of 44% protein and with good ratios of essential amino acids [10, 11, 16, 17]. It is known for its high fat content, with high composition of essential fatty acids, up to 16% of α-linolenic acid [11]. *Senene* is also rich in vitamins and minerals with higher nutrients bioavailability compared to most plants; up to 2.12 mg/g vitamin A, 0.99 mg/100 g folate, 2 to 16.6 mg/100 g iron and 17 mg/100 g zinc have been reported [5].

A few studies on *R. differens* from East Africa, mainly Uganda and Kenya, have reported data on trade and nutritional potential with fewer studies from Tanzania reporting on biology and phenology [5, 13, 15]. There are even fewer studies on culture, beliefs and indigenous technology associated with *R. differens*, despite the rising acceptance and prominence of this delicacy. It is crucial to understand indigenous technologies, culture and beliefs associated with consumption of *R. differens* for scaling up commercialization innovations as well as designing adoption for ongoing scientific findings.

## Methods

### Study area

The study was conducted in the northwestern corner of Tanzania in Kagera region, lying between 1°00′ and 2°45′S and 32°40′E. The capital town of Kagera is Bukoba, which is about 1500 km from Dar es Salaam by road. Kagera region shares borders with Uganda to the North, Rwanda and Burundi to the West and Lake Victoria to the east. Main economic activities in this area include farming, fishing, livestock keeping and mining [18]. The natives of Kagera are mainly of *Haya* tribe for whom *senene* is an esteemed delicacy. This hilly terrain region with thick tropical vegetation including forests and wide-open grasslands experiences two rain seasons. The long rain season lasts from March to May, during which the study was conducted, while the short rain season lasts from October to December. The study focused on Muleba and Kagera urban districts where *senene* harvesting and enterprise is common.

### Sampling frame, sampling and interviews

Twenty five males and 26 females randomly selected *Haya* adults from Muleba and Kagera urban districts of Kagera region were interviewed. Seven male and three female elders of *Haya* tribe and *senene* collectors were selected using snow ball sampling due to their distinct knowledge of local culture and *senene*. *Senene* collectors who were found at the *senene* collection points during the night were also interviewed. These experts were interviewed mainly to validate the findings and provide additional clarification around some of some beliefs and indigenous technologies outlined by interviewees. Information was collected through face to face interviews using questionnaires administered in Swahili. The study was of an ethnographic nature with interviews focused on perceptions, cultures and beliefs, indigenous technologies in harvesting, processing and preservation, and shelf-life as well as traditions towards *senene* consumption. *Senene* prices and nutrition knowledge among *senene* consumers were also collected through the questionnaires. In addition to the interviews, observations on harvesting, cooking and traditional processing of *senene* was carried out at the homestead, farms and wild fields. To document some of the traditional practices, photos of the insects, traditional traps and *senene* markets were taken. Samples of *senene* were collected from the fields and markets for identification and inventorying.

### Data analysis

Data were coded and entered in Statistical Package for Social Sciences (SPSS) version 21 where descriptive statistics were computed.

## Results and discussion

### Social-economic information of the respondents

Data was collected on socio-economic characteristics of respondents involved in the study including age, gender, tribe and occupation. The age of the respondents ranged from 21 to 60 years old with a majority (43%) aged 21 to 30 years. Fifty one percent of respondents were female, these were first-hand informers as *senene* collection, and processing is dominated by women. Edible insect consumption is influenced by cultural belief system and differs across different ethnic groups, 90% respondents were Hayas, other came from the *Sukuma*, *Nyambo*, *Bunganda* and *Kerewe* tribes. Interviewees’ occupations were mainly local government officials (39%) mostly from nature and forestry department, self-employed in informal sector including *senene* traders (33%), peasant farmers (15%) and students (13%).

### Varieties and seasonality

**Fig. 1:**
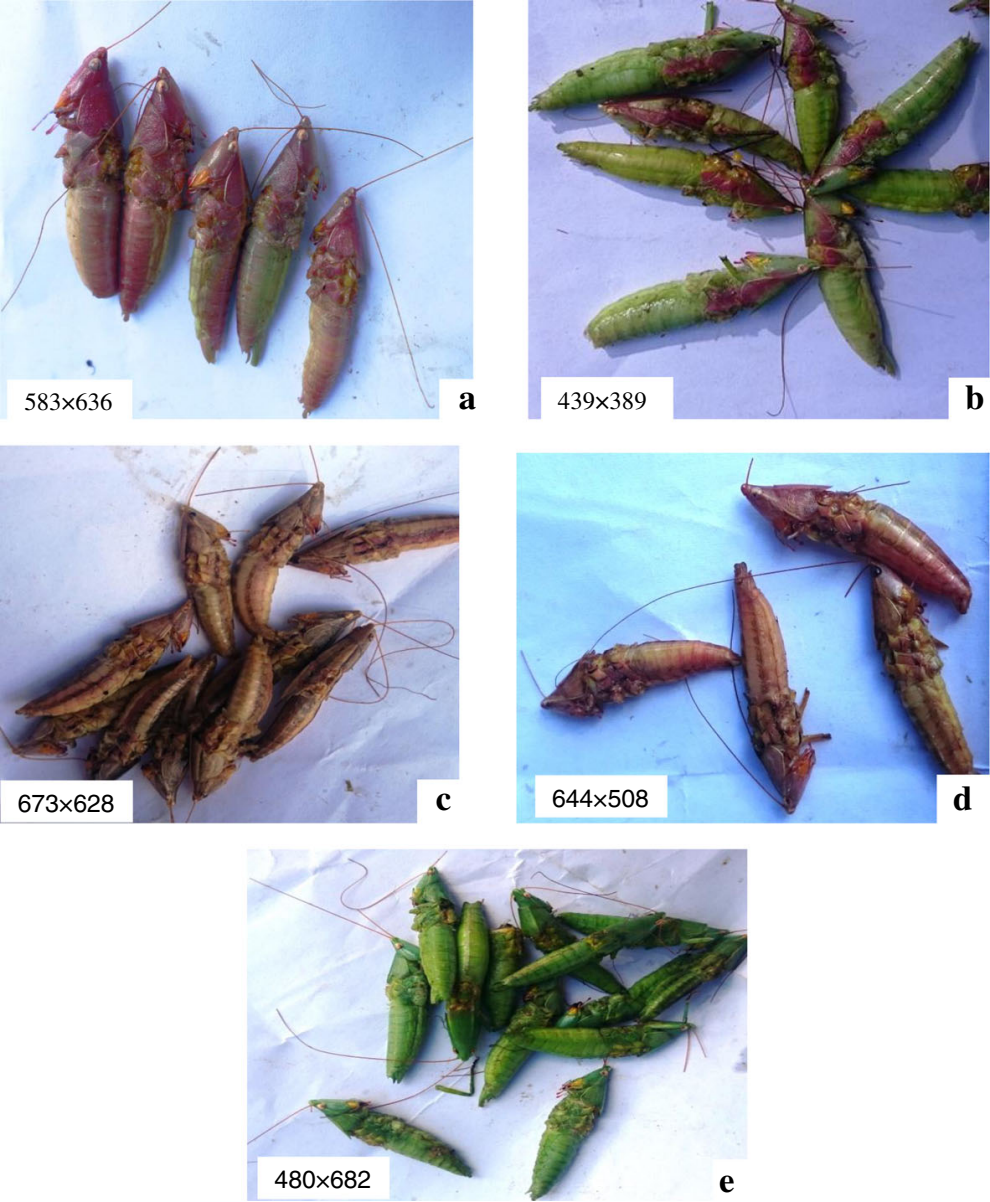
**a**–**e** De-winged and de-legged *senene* varieties found in Kagera as identified by *Haya* ethnic group. **a** *Mwanamwana* meaning beautiful woman. **b** *Mfaume* meaning a ruler or confused person. **c** *Kishorowanda* meaning colourful bird. **d** *Katikomile* meaning dry tree. **e** *Kimbisimbisi* meaning greeny

The respondents identified five culturally significant forms of *senene*, each carrying meaningful and unique *Haya* name (Fig. 1 a–e.). The names were based on the appearance and behaviour of each. Purple coloured *senene* were known as *mwanamwana*, which means ‘beautiful woman’ in *Haya* language. This was the rarest form of all. *Kishorowanda* is green with purple stripes. *Mfaume* is brown in colour, and it was said to be the most unpredictable, fierce, biting and hard to trap. *Katikomile* means dry tree in *Haya language* and was a brown-khaki *senene*; it was said to signify the end of the *senene* season. *Kimbisimbisi* was the name given to common green *senene* meaning ‘green tree leaves’, most abundant during the *senene* season. Studies on morphology report six colour forms which are said to be genetically controlled [9, 12]. Some natives suggested that the varieties taste different with, *mwanamwana* being reported as the tastiest. The green form was associated with femaleness and richness of the season while brown was said to mark the end of the season and was associated with maleness*.*

*Senene* swarms twice a year: April to June being the longest season and November to December being characterised by high numbers of *senene*. This season reaches its peak on around 9th in December, which is also an important national holiday in Tanzania known as *Jamhuri day.* It was believed that if someone could not succeed in collecting *senene* on this day, they are unlucky. Small black-brown flies known as Nairobi fly (*Paederus eximius* and *Paederus sabaeus*) accompanies *senene*, these tend to cause swellings on the faces and arms of the collectors a common challenge faced by *senene* collectors. ‘Gathering senene is not an easy job as senene do come with soldiers’ said one respondent who is also a *senene* collector when asked about the challenge he faces during collection. The feeling is mutual to *senene* collectors in Uganda as reported by Agea [13].

### Occurrence and availability

When asked about the occurrence of *senene*, one old man aged 72 years explained that ‘it is just God’s plan for Kagera, I don’t even understand because they just drop from heaven’. Most natives believe that *senene* came from heaven just like *Manna* dropped to Israelites in biblical times according to the Holy Bible in Exodus 16:1–36. It was further suggested that *senene* came from Lake Victoria; few believed that *senene* emerged from bushes and pine trees while others said they came from dense-dark clouds. According to Matojo and Yarro [19], bushes are the breeding grounds for *R. differens*, where both swarming and non-swarming adults lay eggs in ribbons cemented together. Time lapse between oviposition and maturity is reported to take about 3 months [14]. *Senene* swarms are reported to have decreased over time; some key informers’ suggesting that climate change has significantly affected the current volumes of *senene*.

### Traditions, consumer perception, custom and taboos

‘Senene is an icon of Kagera, God’s gift from heaven for Haya’ as reported by one retired forest officer from Bukoba Municipal Council*.* It is a delicacy normally reserved for men and in-laws. Respondents listed different reasons for consumption; the most common of which include respect of traditional delicious delicacy (44%) and source of nutrients (41%) and tasty nature of *senene* as multipurpose sauce (15%).

*Senene* is a delicacy exported to *Haya* relatives living in distant places like UK or to wedding ceremonies held as far as Dar es Salaam, 1500 km from Bukoba. ‘Haya wedding ceremony will not be perfect until guests are welcomed by a pack of this delicacy at the entrance’, said a respondent. According to *Haya* traditions, a reception with a plate of *senene* is a symbol of respect and acceptance to that family. *Senene* remains a protected snack, not to be offered to any person but few esteemed individuals.

Eating raw *senene* was reported to be a prohibited act; 92% of respondents had never eaten raw *senene*. Eating them raw was regarded unethical by most respondents who associate it with stomachache (52%) and diarrhoea (48%). There is scientific evidence that raw insects contain higher microbial counts, thus consuming them raw can bring such complications [20]. The remaining 8% had eaten fresh raw *senene* out of greed, although some apparently appreciated the raw *senene.* A young male respondent said ‘senene are just so delicious even when freshly raw’*.* S*enene* are used in preparation of snacks, rat bait, chicken and fish feed; birds and monkeys are also reported to be consuming this important *haya*’s delicacy.

Respondents reported that as it is with many African taboos, women and children had limitations towards s*enene* consumption. Pregnant women were prohibited from eating *senene* or they would give birth to children with coned-head like that of *senene*. It was further believed that giving *senene* to infants and young children would make them unable to speak for the rest of their lives. This taboo is similar to that of the grasshopper clan of the Baganda in Uganda where women are prohibited to eat *senene* though they are allowed to catch them and cook for their husbands [13, 21]. However, several key informants questioned the taboo, suggesting the taboo was due to men’s selfishness and desire to have all *senene* made for them. No medicinal beliefs were reported though *senene* are thought to be an aphrodisiac. There was a widespread belief that marriages became stronger and happier during *senene* season as women proudly collected and prepared them for their husbands, and, in return, they receive gifts such as *kitenge*, an esteemed traditional piece of cloth. *Senene* appendages, ovipositor and wings are not generally consumed and are discarded far from the main house across the road junction, as a show off to neighbours. Key informants explained the basis of this belief was to avoid the rotten smell, and prevent ants and flies from disturbing them in the house.

Although studies report protein rich arthropods, such as shellfish (mainly shrimp, lobster and crayfish) to be widely known for their ability to induce allergic reactions [22, 23], respondents reported no allergy caused by consumption of *senene*. Few respondents (28%) reported diarrhoea cases after consuming excessive amount of *senene*; probably due to the high fat content and poor hygienic practices during processing.

### Collection and harvesting

**Fig. 2:**
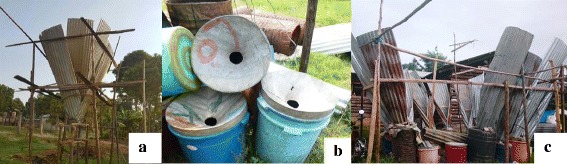
*Senene* harvesting using traditionally made traps. **a** Corrugated iron sheets folded into a cone shape and held in position using props. **b** Large buckets with a hole on the lid. **c** An assembled trap ready for harvesting

**Fig. 3:**
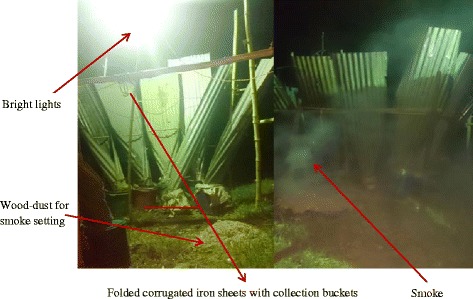
*Senene* harvesting trap during the night

The findings of this study suggested that traditionally, harvesting of fresh raw *senene* for family consumption was done through wild collection in fields. However, nowadays, it is common to harvest *senene* at home under very bright light since *senene* are nocturnal. Homestead collections were normally done by women and children of school age early in the morning before sunrise when they are inactive and hence easily captured. Commercialization of *senene* has triggered advancement in harvesting technology, using locally made traps (Figs. 2c and 3). The traps were made using folded iron sheets, large buckets and three very bright light bulbs (400 W each) as shown in Fig. 3.

The iron sheets were folded to a cone shape leading to the large bucket, which collected the falling *senene* (Fig. 2b). During the night, smoke was set under the bright light, which confused the *senene* hampering their ability to fly. A key informant reported that “Like a cow moving its head to the abattoir unsuspectingly, senene followed the bright light and dropped on folded iron sheets which slid them directly to the large bucket where they could not move out”. When the buckets were full, trapped *senene* were transferred to big sisal sacks or bags and the traps reset ready for the next harvest until the swarm stopped, usually around 5 am during high seasons. *Senene* collectors reported that during high seasons normally eight to ten sacks (one sack approximately100 kg capacity) were collected on a single night swarm.

The majority of the respondents reported that they obtain *senene* for their consumption from market purchase (43%) while others from wild collection (31%), from setting traps (18%) and as a gift from relatives (8%).

### Processing methods

**Fig. 4:**
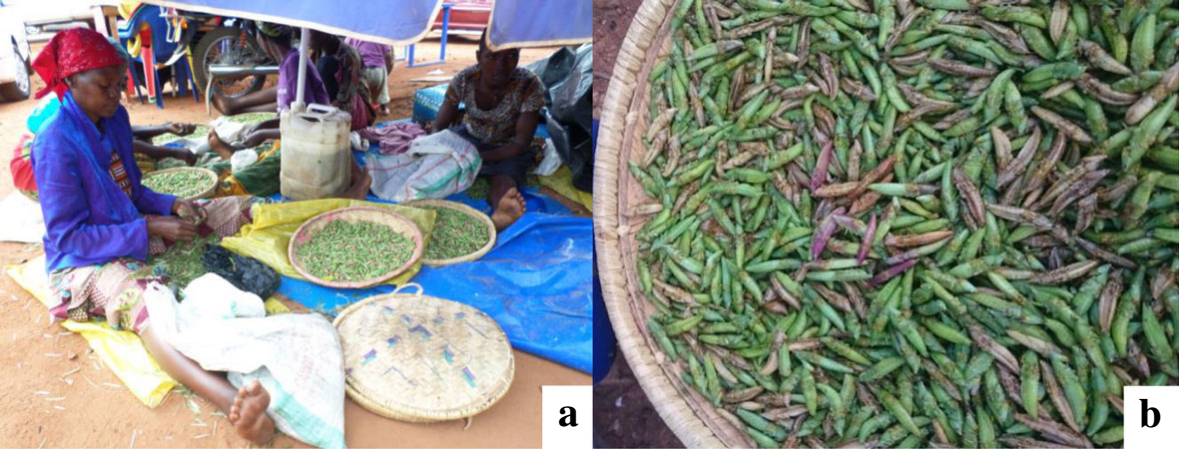
**a** *Senene* cleaning and sorting at Bukoba town *senene* market. **b** Cleaned *senene* ready for sale

**Fig. 5:**
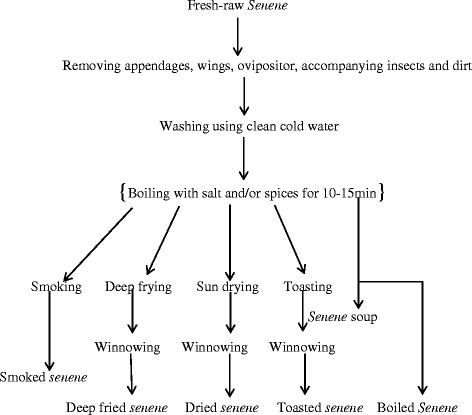
Fresh *senene* processing flow diagram

From observations and responses from respondents, different processing methods have been grouped and discussed in different stages as shown in Figs. 4 and 5.

Edible insects processing is a common practice in almost all areas where they are consumed [24, 25]. Traditional processing methods are mostly similar among insect-consuming societies in Africa with slight differences between the processes. Processing is normally done for value addition to increase palatability, safety and for preservation purpose. Processing ensures removal/reduction of anti-nutrients such as phytates and tannins [26]. Processing methods significantly impact the nutritional value of the insects [10].

### Cleaning and washing of fresh raw *senene* after harvest

Cleaning was done to remove inedible body parts namely wings, appendages and ovipositor for female *senene* (Fig. 4). Wood ashes are used to increase friction and ease the process. Antennae, which are slippery and light, were often not removed as they disappeared within processing chain. At this stage, other insects collected with the *senene* as well as grasses and other waste materials are removed. Some reported washing with cold water before further processing; however, some did not wash claiming that washing would drain out fat and make *senene* less enjoyable.

### Boiling

Cleaned and/or washed *senene* were placed in boiling salted water with or without spices (such as onions, garlic, ginger and cardamom) and then left to boil for about 15 min (Fig. 5). This is normally a stage in making smoked, toasted and deep fried s*enene.* However, boiled *senene* can also be eaten, either drained out or consumed with the stock. The soup (liquid part without *senene*) is sometimes mixed with boiled banana and given to children by some of the locals (10%).

### Smoking

**Fig. 6:**
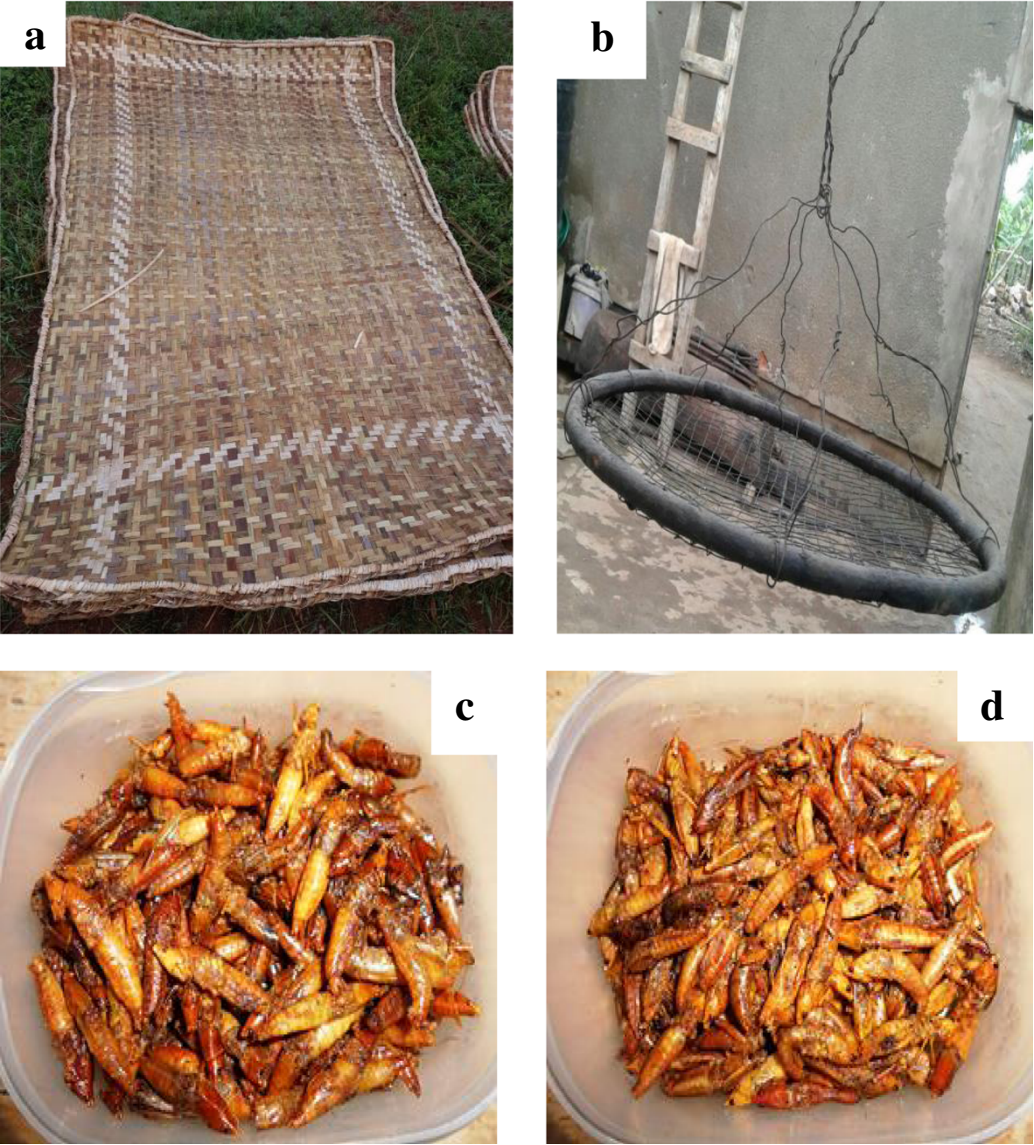
**a** *Ebigara*. **b** *Okashelo*. **c** Deep fried *senene*. **d** Toasted *senene*

**Fig. 7:**
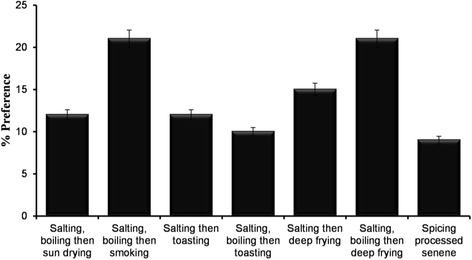
Response on consumer preferences in relation to *senene* processing method

This was reported to be a traditional method of processing *senene.* Results showed that 21% of *senene* consumer preferred smoked *senene* above other processing methods, especially older people (51–60 years) (Fig. 7). The smoky aroma of smoked *senene* and its antique was among the mentioned reasons for its acceptance. Fresh raw or boiled *senene* are rolled in fresh banana leaves then placed on the kitchen roof (*obutala* in *Haya* language). Others used a special roof extension for *senene* placed along the direction of smoke from the burning firewood known as *akashelo* (Fig. 6b). Smoking was explained to follow two similar processing lines as shown in Figs. 4 and 5. Boiling was reported to be a mandatory step for short-time (2–4 days) smoked *senene*; it was optional where *senene* were to be smoked for a longer period. *Ekyangwe* was the name for special leaves burned together with firewood for *senene* smoking. The firewood used for *senene* smoking were those from trees characterised with slight or no smell, thus preventing alteration of *senene’s* natural flavour.‘You don’t just smoke senene because there is smoke’ said a key informant when asked about the type of trees used for *senene* smoking. Eucalyptus, cassava stems (*Manihot esculenta*), umbrella tree (*Maesopsis eminii*) and traditional trees such as *msila* were reported to have the best firewood for *senene* smoking.

### Toasting

As summarised in Fig. 5, toasting is done by placing fresh raw or boiled *senene* in a hot pan, stirring them until they turned brown with a meat-like smell. Toasting was among the oldest methods still being practiced. Toasted *senene* became crunchy with less oil; thus, one could eat plentiful amounts before satiated. Toasted *senene* are brownish in colour (Fig. 6c). In urban areas, where the technology is available, baking ovens are used to toast *senene.*

### Deep frying

Deep frying is currently the most (22%) used and preferred processing method of processing *senene* by younger people (Fig. 7). Cleaned boiled or fresh raw *senene* were placed in boiling cooking oil (cottonseed oil was common) for about 10 min until they turned deep-brownish (Fig. 6d). The simplicity of this method has made it common and most liked among *senene* traders.

### Sun drying

Sun drying was mainly for preserving *senene* for future processing, followed by storage in sisal sacks or plastic buckets. *Senene* became ready for consumption within 2 days of sun drying. The process took place on a locally made mat known as *ebigara* in *Haya* language (Fig. 6a).

### Preservation and shelf-life

**Fig. 8:**
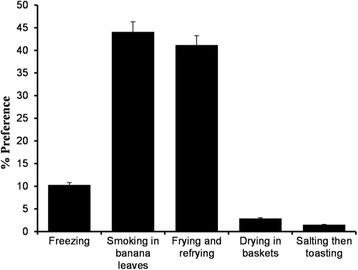
Response on consumer preferences in relation to *senene* preservation methods

Fresh *senene* have a very short shelf-life of about 12 to 48 h at room temperature of 24–28 °C. Fresh raw *senene* were normally stored in polyethylene, sisal bags or mesh cloth bags that allow air to pass through. *Senene* traders normally used polyethylene bags to transport fresh *senene*; some spread *senene* on a mat temporarily before further processing. For a longer shelf-life, fresh *senene* are preserved using several traditional techniques such as smoking above the cooking place (44%) and sun drying (Fig. 8). Nowadays, re-frying is common (41%), and deep freezers and refrigerators are also used for storage and preservation. The average reported shelf-life for traditionally preserved *senene* is 12 months.

### Potential of *senene* in addressing child malnutrition

Use of *R. differens* in enriching currently used complementary foods in this region, namely boiled banana (32%) and cereal porridge (52%), can make a considerable contribution in reducing stunting rates. The Kagera region is named among the three regions exceeding the WHO 40% threshold of severe stunting [27]. Apart from the associated taboos towards withholding *senene* from children, the majority of the respondents reacted positively on the issue of including it in the infants’ and children’s diets. Table 1 presents respondents’ feelings and suggestions concerning giving children *senene*.

**Table 1:** Responses on feeding children with *Senene*

Information collected	Percentage respondents (%)
Population currently giving *senene* to children (*n* = 51)	
Yes	76.0
No	24.0
Reasons for feeding children *senene* (*n* = 43)	
Are nutritious	65.1
Children like *senene*	27.9
I just feed them	7.0
Reasons for not feeding children *senene* (*n* = 18)	
They cannot digest *senene*	11.1
They cannot chew	33.3
They may choke and block air	38.9
It is a taboo	16.7
Readiness to give *senene* to children if well prepared (*n* = 51)	
Yes	90.0
No	10.0
Commonly consumed complementary foods (*n* = 59)	
Single cereal	39.0
Mixed cereals	13.6
Boiled banana	32.2
Mixed cereals and legumes	8.5
Eat what adults eat	6.8
Opinions on using *senene* in complementary feeding (*n* = 116)	
It is a nutritious food	63.8
They should be given only *senene* soup	6.9
*Senene* should be ground and sieved for children to swallow	13.8
More research to be done	6.0
Heads should be removed before giving to children	3.4
Not a good thing because children will not be able to talk	2.6
It is an act of disrespect to give *senene* to children	1.7
Children likes *senene*	1.7

The use of edible insects in addressing malnutrition has been evaluated by several researchers [28–34]. Silkworm pupae were included among essential ingredients for supplementary food to malnourished children in Congo [31]. Edible insects have also been utilised to enrich plant-based complementary foods. In Kenya, winged termites were used to enrich amaranth-based complementary foods [32] while in Cambodia, edible spiders were utilised to enrich a rice-based complementary food [28].

### *Senene* trade

**Fig. 9:**
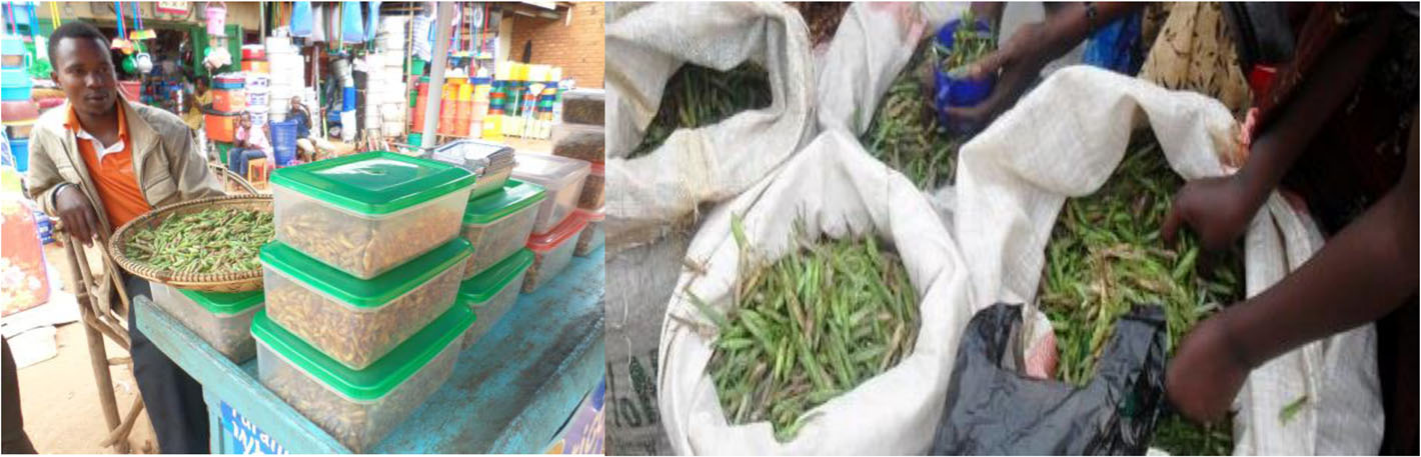
*Senene* trade at Bukoba town *senene* market

In Kagera region, *senene* trade was carried out by both men and women, with men dominating the commercial collection end while women were responsible for cleaning and processing (Fig. 4). Trade starts within the Kagera region among *Hayas* as 43% of respondents reported to obtain their *senene* from local traders. Both processed (mostly deep fried) in some cases, fresh raw *senene* were largely sold in this market (Fig. 9). Deep fried *senene* were transported throughout Tanzania; Dar es Salaam is the main destination. The price of fresh *senene* during the season averaged Tsh 1534.88 per kilogram while that of deep fried *senene* averaged Tsh 4254.05 per kilogram. Smoked, sundried and toasted types were not commonly found at the market place. The price of processed *senene* was more than twice that of fresh *senene* mainly due to tedious and laborious process of removing wings, appendages and ovipositor. *Senene* business is widely carried out from hawkers selling locally packed *senene* along the road side to big stores and supermarkets selling processed *senene* across Tanzania. Contribution to household income has not been reported, but most traders report that it covers almost half of their basic needs including school fees. Sixty one percent of respondents reported that one of the potential uses of *senene* is for income. There is potential for women employment as cleaning, and processing tasks are assigned to women, who are able to work to generate an income from *senene* production for about 3 months a year.

In Uganda, the grasshopper trade is characterised by wholesalers who buy grasshoppers from collectors and sell to retailers, who sell to consumers around Kampala [13]. The edible insects business is growing globally as the market for edible insects collected in the wild as well as reared in captivity increases [33]. For example, Belgium annually imports up to three tonnes and France five tonnes of dried mopane caterpillar from the Democratic Republic of Congo and Zambia [9, 29].

## Conclusion

Our study shows that *R. differens* represents an important edible insect species for East African societies around the lake zone. Traditions, cultures and beliefs of different societies highly influence dietary choices and adoptions of new products. Understanding *senene*’s ancient processing methods and indigenous technologies play a crucial role in innovations involving the food industry. Wild fields such as bushes and short grasses are important habitants for the life cycle of this seasonal delicacy. Maintenance of food habits and their values, in connection with urban migration, are main drivers of the increasing trade in edible insects across East Africa. *Senene* carries economic potential, essential for improving incomes and livelihoods of women and smallholder farmers. *Senene* can also be of use in enriching plant-based complementary foods for improved nutrition status of children.

## Recommendations

As early as 1975, edible insects were advocated as a food item to stave off malnutrition [3], but in order to get maximum benefit from *senene* as a food item, improvements to the indigenous processing technology are required to assure quality and safety of marketed *senene*. Promotion of *senene* as a delicious food among all consumers should be encouraged in order to tackle the challenges posed by limiting taboos. Inclusion of *senene* in the diets of infants and young children is a subject which requires attention of the researchers. Studies on environmental friendly ways to raise *senene* in captivity will increase the year round availability of this currently seasonal delicacy. Shelf-life studies on the *senene* processed by different methods are needed.

## Abbreviations

DRC: Democratic Republic of Congo
SPSS: Statistical Package for Social Sciences
